# Human and Rodent Skeletal Muscles Express Angiotensin II Type 1 Receptors

**DOI:** 10.3390/cells9071688

**Published:** 2020-07-14

**Authors:** Rafael Deminice, Hayden Hyatt, Toshinori Yoshihara, Mustafa Ozdemir, Branden Nguyen, Sanford Levine, Scott Powers

**Affiliations:** 1 Department of Applied Physiology and Kinesiology, University of Florida, Gainesville, FL 32608 USA; rdeminice@uel.br (R.D.); t-yoshih@juntendo.ac.jp (T.Y.); ozdemirm@ufl.edu (M.O.); branden.nguyen@ufl.edu (B.N.); spowers@hhp.ufl.edu (S.P.); 2 Department of Physical Education, State University of Londrina, Londrina 860570-970, Brazil; 3 Department of Exercise Physiology, Juntendo University, Chiba 270-1695, Japan; 4 Department of Surgery, University of Pennsylvania, Philadelphia, PA 19104, USA; sdlevine@mail.med.upenn.edu

**Keywords:** renin angiotensin system, muscle atrophy, mechanical ventilation, diaphragm, muscle wasting

## Abstract

Abundant evidence reveals that activation of the renin-angiotensin system promotes skeletal muscle atrophy in several conditions including congestive heart failure, chronic kidney disease, and prolonged mechanical ventilation. However, controversy exists about whether circulating angiotensin II (AngII) promotes skeletal muscle atrophy by direct or indirect effects; the centerpiece of this debate is the issue of whether skeletal muscle fibers express AngII type 1 receptors (AT1Rs). While some investigators assert that skeletal muscle expresses AT1Rs, others argue that skeletal muscle fibers do not contain AT1Rs. These discordant findings in the literature are likely the result of study design flaws and additional research using a rigorous experimental approach is required to resolve this issue. We tested the hypothesis that AT1Rs are expressed in both human and rat skeletal muscle fibers. Our premise was tested using a rigorous, multi-technique experimental design. First, we established both the location and abundance of AT1Rs on human and rat skeletal muscle fibers by means of an AngII ligand-binding assay. Second, using a new and highly selective AT1R antibody, we carried out Western blotting and determined the abundance of AT1R protein within isolated single muscle fibers from humans and rats. Finally, we confirmed the presence of AT1R mRNA in isolated single muscle fibers from rats. Our results support the hypothesis that AT1Rs are present in both human and rat skeletal muscle fibers. Moreover, our experiments provide the first evidence that AT1Rs are more abundant in fast, type II muscle fibers as compared with slow, type I fibers. Together, these discoveries provide the foundation for an improved understanding of the mechanism(s) responsible for AngII-induced skeletal muscle atrophy.

## 1. Introduction

The renin-angiotensin system (RAS) is well known for its role in the regulation of blood pressure, fluid homeostasis, and electrolyte balance. Importantly, compelling evidence reveals that increased RAS signaling promotes skeletal muscle atrophy (reviewed in [1,2,3,4]). Indeed, congestive heart failure, chronic kidney disease, and prolonged bed rest are all conditions associated with skeletal muscle atrophy resulting from elevated circulating angiotensin II (AngII) [5,6,7,8,9]. 

In theory, high plasma levels of AngII can promote skeletal muscle atrophy by indirect or direct effects on skeletal muscle fibers. For example, AngII can indirectly foster muscle atrophy by increasing circulating glucocorticoids (i.e., cortisol), interleukin-6 (IL-6), or serum amyloid A (SAA). Indeed, elevated plasma levels of cortisol, IL-6, or SAA can independently, or collectively, stimulate skeletal muscle atrophy [10]. In particular, elevated cortisol promotes muscle atrophy by increasing proteolysis and decreasing muscle protein synthesis [11]. Furthermore, high concentrations of IL-6 and SAA act synergistically to evoke muscle atrophy by depressing Akt/mTOR signaling resulting in decreased muscle protein synthesis and accelerated proteolysis [10]. 

In addition to these indirect actions, high circulating AngII can directly stimulate muscle atrophy by binding to angiotensin II type I receptors (AT1Rs) on the sarcolemma of muscle fibers. This direct stimulation of AT1R signaling in muscle fibers activates the predominant NADPH oxidase isoform (NADPH oxidase 2, NOX2) in skeletal muscle culminating in superoxide production, increased proteolysis, and fiber atrophy [3,12]. 

Although it is feasible that circulating AngII can act directly to promote skeletal muscle atrophy, controversy exists about whether skeletal muscle fibers express AT1Rs. For example, one camp of investigators contend that skeletal muscle does not express AT1Rs and that AngII promotes fiber atrophy by indirect effects alone [4,10,13]. In contrast, other investigators argue that skeletal muscle fibers express AT1Rs and that AngII stimulates muscle atrophy by directly binding to AT1Rs on the sarcolemma [14,15,16,17,18,19]. These discordant findings are likely the result of limitations in previous studies. For example, most reports have used commercially available antibodies that exhibited nonspecific binding [20,21]. Furthermore, several studies have reported the abundance of AT1Rs within whole muscle homogenate that is contaminated with other tissues that express AT1Rs (e.g., blood vessels). Moreover, several investigations have measured AT1R abundance in myotubes that did not reflect the number of AT1Rs within adult skeletal muscle fibers. Hence, experiments that avoid these shortcomings are required to establish if AT1Rs are expressed in adult skeletal muscle fibers. Therefore, using a rigorous experimental approach, we tested the hypothesis that AT1Rs are expressed in both human and rat skeletal muscles. This postulate was carefully tested using a multi-technique approach. First, we established both the location and abundance of AT1Rs on skeletal muscle fibers using an AngII ligand-binding assay. Second, using a new and highly selective AT1R antibody, we carried out Western blotting to determine the abundance of AT1R protein within isolated single muscle fibers. Finally, we confirmed the presence of AT1R mRNA in isolated single muscle fibers from rodents. Collectively, our results reveal that AT1Rs are present in both human and rodent skeletal muscle and that AT1Rs are differentially expressed across different rodent skeletal muscle fiber types. 

## 2. Materials and Methods

### 2.1. Institutional Approval for Experiments

The protocol for human muscle biopsies was approved by the University of Pennsylvania institutional review board (IRB protocol ID: 802015, 2007). All biopsy specimens were obtained with appropriate written informed consent. All animal experimental procedures were performed in accordance with the Institute of Animal Care and Use Committee at the University of Florida (IACUC protocol ID: 201810432, 2018). 

### 2.2. Human Diaphragm Biopsies

Human diaphragm muscle biopsies were obtained from 6 patients (3 female, 3 male, ages 18–58) undergoing surgery for either benign lesions or stage 1 lung cancer. Full-thickness biopsy specimens were removed from the right anterior costal diaphragm. Muscle samples were rapidly frozen in isopentane, cooled to the temperature of liquid nitrogen, and stored at −80 °C until assay. 

### 2.3. Animal Muscle Dissections

Female Sprague-Dawley rats (~4 months old, *n* = 9) were used in this study. Animals were housed at the University of Florida Animal Care Service Center and maintained at a 12:12 h light–dark cycle at a mean temperature of 22 °C with ad libitum access to food and water. Following animal sacrifice, the descending aorta, kidneys, diaphragm, soleus, and plantaris muscles were dissected. Tissues were immediately frozen in isopentane, cooled to the temperature of liquid nitrogen, and stored at −80 °C for subsequent analysis.

### 2.4. Experimental Approach

To test the hypothesis that skeletal muscle fibers express AT1Rs, we studied both respiratory and locomotor skeletal muscles and used a multi-technique approach. For example, to determine if skeletal muscle express AT1Rs, we isolated individual muscle fibers from both the human diaphragm and three skeletal muscles in the rat (i.e., diaphragm, soleus, and plantaris). The diaphragm was selected for analysis in both humans and rodents because of recent reports demonstrating that RAS signaling plays a key role in ventilator-induced diaphragm atrophy [22,23]. No gender differences exist in the rate or patterns of skeletal muscle atrophy during hindlimb immobilization or the diaphragmatic atrophy that occurs during prolonged mechanical ventilation. Therefore, female rats were arbitrarily selected for study in these experiments. The rat soleus and plantaris muscles were studied because these rodent hindlimb locomotor muscles atrophy in response to atrophic stimuli and these muscles differ markedly in their fiber type composition; the soleus muscle in rats contains primarily slow, type I fibers, whereas the plantaris muscle is dominated by fast, type II fibers [24]. The rat aorta and kidneys were examined for comparative purposes because these tissues are known to contain large numbers of AT1Rs. 

To determine the presence of AT1Rs in muscle fibers, we used a three-pronged experimental approach as follows: (1) Immunoblotting, using a highly selective antibody, to determine the presence of AT1Rs in single muscle fibers; (2) identification of AT1Rs on the sarcolemma of skeletal muscle fibers using an AngII binding assay; and (3) determination of AT1R mRNA in isolated single muscle fibers from rats. Details of these experimental procedures follow. 

### 2.5. Immunoblotting

The relative abundance of AT1R protein was determined via Western blot analysis using homogenate from isolated single muscle fibers of human diaphragm and the diaphragm, soleus, and plantaris muscles from rats. Single muscle fiber isolation was performed to eliminate contamination of the homogenate with AT1R protein contained within other tissues (e.g., vasculature) found in bundles of muscle fibers. Specifically, ~100 single muscle fibers (~3 mm long) were isolated under a stereomicroscope (AO569, American Optical, Southbridge, MA, USA) in a (relax) solution containing 100 mM KCl, 20 mM imidazole, 4 mM ATP, 2 mM EGTA, and 7 mM MgCl2 (pH 7.0 adjusted with KOH); isolated fiber were placed into an Eppendorf tube containing Tris-EDTA buffer (5 mM Tris, 5 mM EDTA, and 1% Triton X-100, pH 7.4) and 1:20 vol/vol protease inhibitor cocktail (Sigma-Aldrich, St. Louis, MO, USA). Samples were, then, exposed to 3 freeze/thaw cycles and homogenized with a small pestle. Then, samples were centrifuged at 1500× *g* for 10 min and the supernatant was placed in Laemmli sample buffer (Bio Rad, 1610747, Hercules, CA, USA) containing 5% dithiothreitol. The muscle fiber homogenates were, then, boiled for 5 min. Proteins within the homogenate were separated via polyacrylamide gel electrophoresis, transferred onto a PVDF membrane, and incubated with an AT1R antibody that had recently been validated with both knockout and overexpression animals [25]. The AT1R antibody was a gift of Dr. Kate Murphy (University of Melbourne, Melbourne, Australia). Membranes were incubated with Alexa Fluro 800 IgG secondary, scanned, and analyzed using an infrared imager (LI-COR Bioscience, Lincoln, NE, USA) using Odyssey 2.1 software. All Western blot images were normalized to total protein using REVERT total protein stain (LI-COR Bioscience). Importantly, REVERT total protein staining has been demonstrated to be superior for Western blot normalization as compared with the use of housekeeping proteins [26]. Note that anti-CD31 (Abcam, #ab28364), a biomarker of vascular tissue, was used to ensure that single muscle fiber samples were not contaminated with vascular tissue. Additionally, PAX7 (Developmental Studies Hybridoma Bank, #AB 528428) was used to determine the abundance of satellite cells within the single muscle fiber homogenate. 

### 2.6. Identification of AT1R Using a Fluorescence-Based Binding Assay

A fluorescence-based binding assay for AT1R was performed using methods adapted from [27,28]. Briefly, AngII was conjugated to the fluorophore TAMRA 5(6)-carboxytetramethylrodamine. OCT-embedded vasculature, kidney, diaphragm, soleus, and plantaris muscles were frozen and serially cut in a cryostat (HM550 Cryostat, Thermo Fisher Scientific, Waltham, MA, USA) in 10 µm sections; then, tissue sections were mounted on slides and dried for 1 h, at 4 °C. The sections were, then, preincubated in assay buffer composed of Hanks’ Balanced Salt solution (Thermo Fisher Scientific, #14025076) supplemented with 0.2% bovine serum albumin (BSA), 0.01% bacitracin, 0.002% phenylmethylsulfonyl fluoride (PMSF), and 0.01% 1,10-phenanthroline, for 30 min, in an ice bath. Slides were, then, air-dried for 5 min and incubated with the following conditions: (1) assay buffer containing unlabeled AngII (Sigma-Aldrich, A9525, St. Louis, MO), (2) assay buffer containing 5 μM TAMRA-labeled AngII peptide (AnaSpec, Fremont, Ca, AS-61181), (3) assay buffer containing unlabeled AngII with Losartan, and (4) assay buffer containing TAMRA-labeled AngII with losartan. Slides were incubated in their respective buffer, for 1 h, on ice. Following incubation, sections were quickly rinsed three times with assay buffer, and then washed five times in assay buffer, for 1 min each. Slides were dried and fluorescence was measured on an Axiovert 200 fluorescent microscope (Zeiss, Göttingen, Germany) using a rhodamine excitation fluorescence filter. For AT1R quantification, the auto local thresholding technique [29] was used to quantify relative fluorescence density in the AT1R binding sections using ImageJ software (National Institutes of Health, Rockville, MD, USA). Relative AT1R values were generated by subtracting background fluorescence (the value of threshold fluorescence detected in the presence of losartan and co-incubated with TAMRA-AngII from serially sectioned tissues from the same sample) from total threshold fluorescence (fluorescence detected with TAMRA-AngII without losartan). All measurements were made in triplicate. Sections incubated in AngII without the TAMRA label were used as negative controls. 

Proof-of-concept for this technique was confirmed during preliminary experiments corroborating the existence of AT1Rs in skeletal muscle as compared with other tissues known to express high levels of AT1Rs. Moreover, the concentration of losartan required for maximal blockade of AT1Rs was also determined during pilot studies (see Supplementary Materials, Figure S1). 

Finally, a non-quantitative fluorescent-based binding assay for AT1R was also performed using isolated single fibers of diaphragm, soleus, and plantaris muscles. The objective of this procedure was to confirm that AT1Rs exist on the sarcolemma of muscle fibers. Single muscle fibers were isolated under a stereomicroscope in a relax solution and placed on a microscope slide. The single fibers were, then, cut into three segments (~2 mm long) and each section was incubated in one of the following three different solutions: (1) assay buffer containing unlabeled AngII, (2) assay buffer containing 5 μM TAMRA-labeled AngII peptide, or (3) assay buffer containing TAMRA-labeled AngII with 1 mM losartan. Solution preparation, incubation, and wash procedures were the same as described previously for tissue sections.

### 2.7. AT1R mRNA 

Because of limited tissue availability of the human diaphragm samples, AT1R mRNA levels were measured in rat skeletal muscles only. To determine the AT1R (*Agtr1a*) mRNA levels in single fibers from plantaris, soleus, and diaphragm muscles, bundles of muscle fibers were placed in RNAlater-ICE for overnight, at −20 °C. The fiber bundles were, then, washed in RNAlater and placed in relax buffer (100 mM KCl, 20 mM imidazole, 2 mM EGTA, 4 mM ATP, 7 mM MgCl2, pH 7.0) on ice. Then, the muscle fibers were isolated from the muscle bundle using fine tweezers under a stereomicroscope (AO569, American Optical). The fibers were freed of connective tissue, vascular, and other debris and ~100 fibers (~3 mm length) were quickly placed into 10 µL of TRIZOL Reagent (Invitrogen, Carlsbad, CA, USA). Immediately after single-fiber isolation, 140 µL TRIZOL Reagent was added (150 µL total solution), the fibers were homogenized using a homogenizer pestle, and then stored at −80 °C until analysis. After 5 min incubation at room temperature, 30 µL of 1-bromo-3-chloropropane was added and tubes were vortexed and incubated for 3 min at room temperature. The mixture was, then, centrifuged at 13,000 × *g* for 15 min (4 °C) and the supernatant collected. For RNA precipitation, 2 µL of glycogen (Thermo Fisher Scientific, Waltham, MA, USA) was added to the supernatant and mixed with 100 µL of isopropanol, incubated at room temperature for 10 min, and at 4 °C for 30 min, and then centrifuged at 13,000× *g* for 45 min. The supernatant was, then, removed, 0.5 mL of ice-cold 75% ethanol was added, and the mixture centrifuged at 13,000 × *g* for 10 min. Pellet wash and centrifugation were repeated once again using 70% ethanol before pellet was dried in air and dissolved in 10 µL of RNase-free water. The concentration and purity (A260/280 and A260/230) of total RNA were determined using a NanoDrop (Thermo Fisher Scientific, ND-2000, Waltham, MA, USA) and reverse transcription was performed with an input of 2.5 μg total RNA using SuperScript VILO MasterMix (Invitrogen).

The PCR conditions used were as follows: 50 °C for 10 min, then 40 cycles at 95 °C for 15 s and 60 °C for 1 min, with a final cycle at 37 °C for 30 s, in a StepOne Plus Real-Time PCR System (Thermo Fischer Scientific). *Agtr1a* (Rn01435427_m1) mRNA levels were quantified using a TaqMan gene expression assay (Thermo Fischer Scientific) and were normalized to *ACTB* (Rn00667869_m1) mRNA levels. Measurements were done in duplicate and the 2^−ΔΔCt^ method was used for data analysis (cycle threshold (Ct) = Ct (gene of interest) − Ct (reference gene)). Relative changes (ΔΔCt) in the expression level of target gene were calculated by subtracting the ΔCt of the diaphragm muscle.

### 2.8. Statistical Analysis

Data are presented as mean ± standard deviation. Comparisons between groups were made by one-way ANOVA, and when appropriate, a Tukey post-hoc test was performed. Significance was established at *p* < 0.05. 

## 3. Results

### 3.1. AT1R Abundance in Rat Skeletal Muscles Determined by AngII-Binding Assay

**Figure 1:**
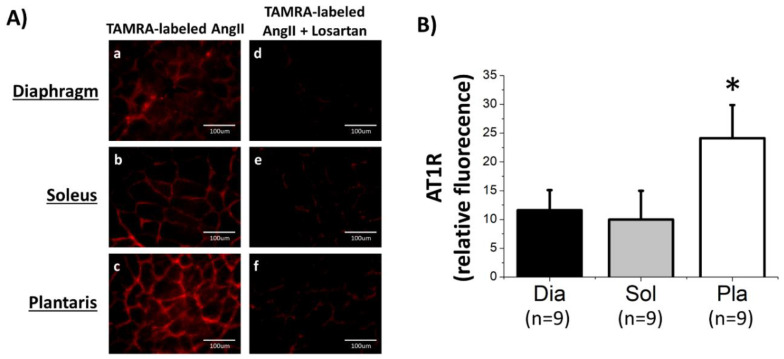
Angiotensin II (AngII) ligand-binding assay reveals the presence and abundance of AT1Rs on the sarcolemma of three rat skeletal muscles. Panel (**A**) contains photographs of TAMRA-labeled AngII binding in muscle cross sections of the diaphragm, soleus, and plantaris muscle. Images a–c represent total AngII binding in each muscle and images d–f depict the nonspecific binding in the presence of losartan; AT1R quantification (Panel (**B**), histogram) was performed by subtracting relative fluorescence of TAMRA-labeled AngII in the presence of losartan (nonspecific binding) from total binding. Data are mean ± SD. * different (*p* < 0.05) from diaphragm (Dia) and from soleus (Sol).

Our experiments tested the hypothesis that human and rat skeletal muscles express AT1Rs. In this regard, note that humans express only one AT1R isoform, whereas rats express two isoforms (labeled as AT1R_A_ and AT1R_B_) [21]; the two AT1R isoforms in rats share 94% identity and are indistinguishable pharmacologically [21]. To determine the abundance of AT1Rs in rat skeletal muscles, we employed three complimentary approaches. First, we utilized a fluorescent-ligand binding assay (TAMRA-labeled AngII) to determine both the location and abundance of AT1Rs in skeletal muscles. Our results reveal that AT1Rs exist in the diaphragm, soleus, and plantaris muscles, as evidenced by a well-defined fluorescence label along the sarcolemma of muscle fibers which agrees with the known location of AT1Rs (Figure 1A (a–c)). In addition, notice that the intensity of the fluorescence was markedly diminished when muscle sections were incubated with losartan to prevent binding of AngII to AT1Rs (Figure 1A (d–f)); this confirms that the fluorescence signal in the muscle was dominated by TAMRA-labeled AngII binding to an AT1R_A_ or AT1R_B_. 

Next, to determine if differences exist in the abundance of AT1Rs between muscles that differ in fiber type composition, we compared the fluorescence levels between cross sections of the diaphragm, soleus, and plantaris muscles. A comparison of the relative fluorescence between these three muscles reveals a higher abundance of AT1Rs within the plantaris muscle as compared with both the soleus and diaphragm muscle (Figure 1B). This is a new and important finding indicating that AT1R abundance is greater in skeletal muscles that contain a high percentage of fast fibers (i.e., plantaris) as compared with muscles comprised of slow fibers (i.e., soleus) or muscles containing a mixture of both slow and fast fibers (i.e., diaphragm).

**Figure 2:**
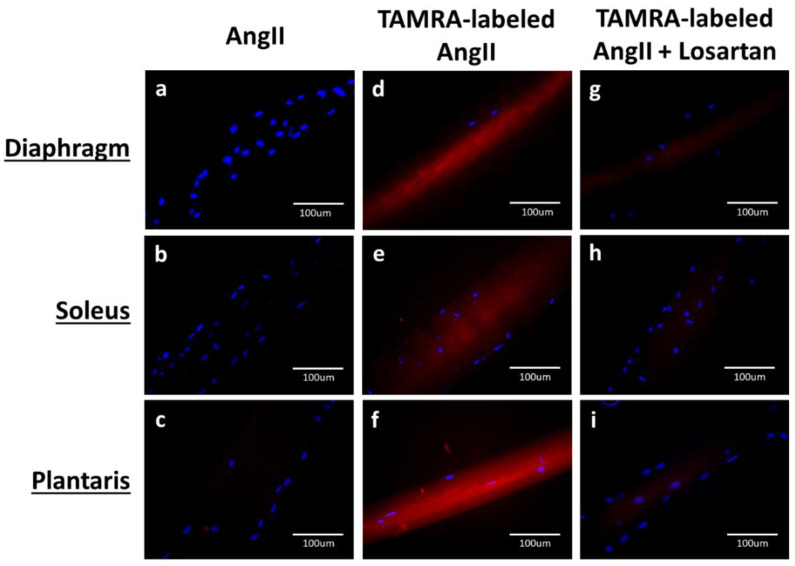
Single fiber histology confirms sarcolemma localization of AT1R in rat skeletal muscles. Figures demonstrate isolated muscle fibers incubated with non-labeled Ang II (**a**–**c**); total binding after incubation with TAMRA-labeled AngII (**d**–**f**); and nonspecific binding after incubation with TAMRA-labeled AngII + 1 mM losartan (**g**–**i**). Skeletal muscle nuclei are identified in blue (DAPI).

Finally, to confirm that the fluorescent signal of AngII ligand binding in the muscle cross sections was not due to AT1Rs in the vasculature, isolated single muscle fibers were also incubated with TAMRA-labeled AngII; these images clearly illustrate the presence of AT1Rs on the sarcolemma of single muscle fibers from the diaphragm, soleus, and plantaris muscles (Figure 2d–f). Note that the fluorescent intensity was absent when single muscle fibers were incubated with non-fluorescent AngII (Figure 2a–c). Importantly, the ligand binding of fluorescent AngII was inhibited by blockade of AT1Rs via losartan (Figure 2g–i). 

### 3.2. Western Blotting to Determine AT1R Abundance in Rat Skeletal Muscles

To provide a second approach to the question of whether AT1Rs are expressed in skeletal muscle, we utilized a recently validated AT1R antibody and performed Western blots on protein homogenates from isolated single muscle fibers [25]. These results corroborate the presence of AT1Rs in skeletal muscle fibers and provide additional support for the hypothesis that skeletal muscles express AT1Rs. To verify that our single fiber homogenates were not contaminated with vascular tissue, we also performed Western blots to determine the presence of the vascular biomarker, CD31. Importantly, CD31 was undetectable in the isolated muscle fiber homogenate (data not shown). Finally, because AT1Rs are present in satellite cells [30], we also assayed the single fiber homogenate for evidence of the satellite cell biomarker, PAX7; notably, PAX7 was not detectable in these homogenates (data not shown).

**Figure 3:**
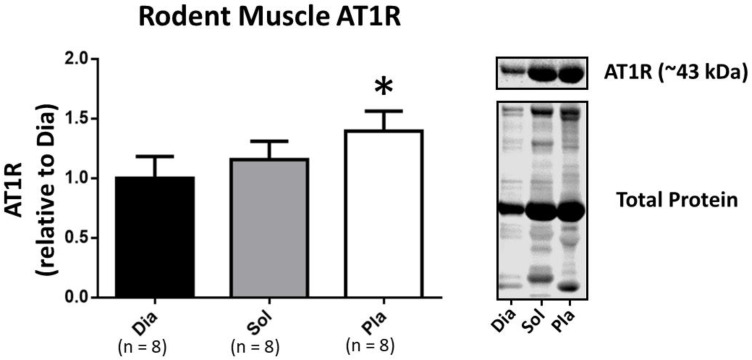
Western blot analysis of isolated single muscle fiber homogenate to quantify the abundance of AT1Rs in the rat diaphragm (Dia), soleus (Sol), and plantaris (Pla) muscles. Data are mean ± SD. * significantly different (*p* < 0.05) from diaphragm (Dia) and from soleus (Sol).

Lastly, our Western blot results also support our findings with the fluorescent AngII-binding assay data indicating that the relative abundance of AT1Rs is greatest in the fast plantaris muscle as compared with muscles comprised of slow, type I muscle fibers (soleus) or muscles containing a mixture of slow and fast fiber types (diaphragm) (Figure 3). 

### 3.3. AT1R mRNA in Rat Skeletal Muscles

**Figure 4:**
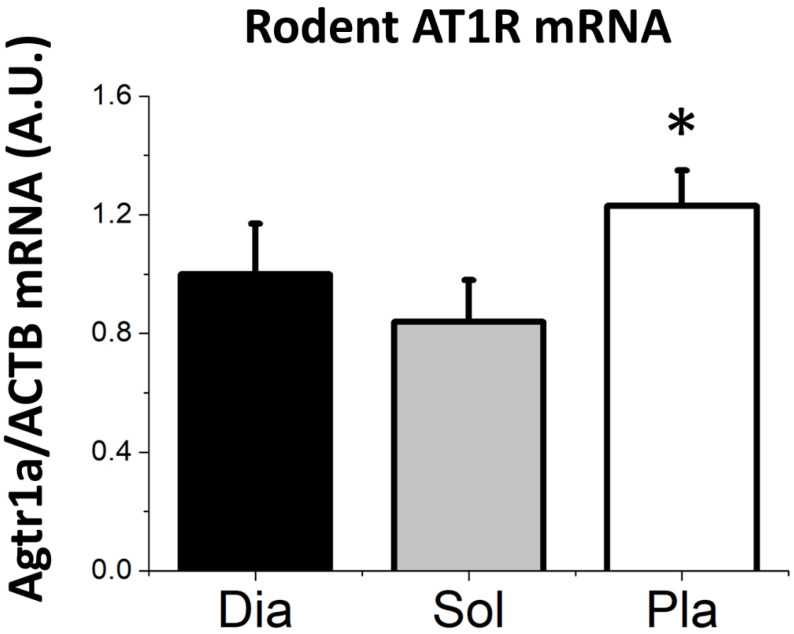
AT1R_A_ mRNA from single muscle fibers isolated from the rat diaphragm (Dia), soleus (Sol), and plantaris (Pla) muscles. Data are mean ± SD. * significantly different (*p* < 0.05) from diaphragm (Dia) and from soleus (Sol).

As a final measure of AT1Ra expression in skeletal muscle, we determined the mRNA levels in single muscle fibers isolated from the diaphragm, soleus, and plantaris muscles. These results reveal that AT1Ra mRNA levels were significantly higher in the fast fiber type, plantaris muscle as compared with both the diaphragm and soleus that contained slower muscle fiber types (Figure 4). 

### 3.4. Both Ligand-Binding and Western Blotting Confirm the Presence of AT1R in Human Diaphragm

**Figure 5:**
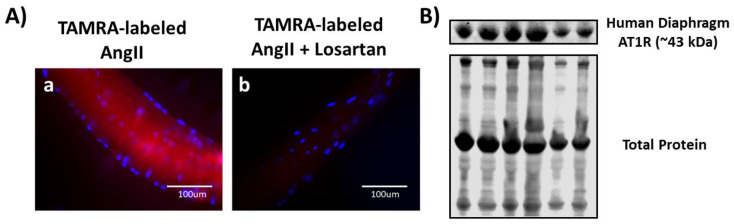
AT1R is present in human diaphragm. Panel (**A**) illustrates isolated muscle fibers from the human diaphragm incubated with TAMRA-labeled AngII (a) and nonspecific binding after incubation with TAMRA-labeled AngII + 1 mM losartan (b). Skeletal muscle nuclei are identified in blue (DAPI); Panel (**B**) illustrates a representative Western blot indicating the presence of AT1R protein in the human diaphragm. This immunoblot was generated from a protein homogenate of isolated single muscle fibers from the human diaphragm.

To establish if AT1Rs are expressed in human skeletal muscle, we performed fluorescent ligand binding on isolated single diaphragm fibers and Western blotting using homogenates of single muscle fibers from human diaphragm muscle biopsies. Similar to the rat diaphragm, the human diaphragm is a mixed fiber type muscle containing both slow and fast fiber types. Identical to our findings in the rat, our results confirm the presence of AT1Rs in isolated muscle fibers from the human diaphragm (Figure 5). Specifically, we observed a prominent fluorescent signal from single fibers incubated with TAMRA-AngII (Figure 5A) and the appearance of a single band at the molecular weight (43 kDa) for AT1R in a Western blot (Figure 5B). Collectively, these results from both rat and human muscle tissue provide robust evidence confirming the presence of AT1R protein in skeletal muscle.

## 4. Discussion

### 4.1. Overview of Major Findings

Using a robust multi-technique approach, our findings support the hypothesis that AT1Rs are expressed in both human and rodent skeletal muscle. Specifically, results from both Western blotting and a fluorescence-based ligand binding assay confirm that AT1Rs are present in both human and rat skeletal muscles. In addition, the presence of AT1R mRNA in rat skeletal muscles provides additional support for this conclusion. These important results provide the first evidence that AT1Rs are expressed in human skeletal muscle. Moreover, our data provide the first robust evidence that AT1Rs exist in rodent skeletal muscles and that AT1Rs are more abundant in fast, type II muscle fibers as compared with slow, type I fibers. Together, these significant discoveries provide the foundation for an improved understanding of the mechanism(s) responsible for AngII-induced skeletal muscle atrophy. A critique of our experimental approach and a discussion of the physiological significance of these findings follows.

### 4.2. Critique of Experimental Approach

Resolving the controversy surrounding the level of AT1R expression in skeletal muscle fibers requires a rigorous experimental approach that avoids the pitfalls associated with previous investigations. In reference to weaknesses of earlier studies, some reports have measured AT1R abundance in only myotubes, whereas others have assessed AT1R levels in whole skeletal muscle homogenate; clearly, both approaches have shortcomings. Furthermore, a major flaw of previous investigations was the use of commercial AT1R antibodies that lacked specificity [20,21]. Indeed, the usage of nonspecific antibodies was likely a key contributor to the lack of reproducibility across the skeletal muscle/AT1R literature. A critical analysis of our experimental approach follows.

To avoid the drawback that myotubes may not be representative of adult skeletal muscle fibers, we measured the abundance of AT1Rs within (adult) human and rat skeletal muscle fibers. Furthermore, to circumvent the shortcoming that whole muscle homogenate contains AT1R from vascular tissue, we measured the AT1R abundance within isolated single muscle fibers. As an added precaution, we confirmed that the homogenate from our single muscle fiber samples did not include the vascular biomarker, CD31.

Because many commercially available AT1R antibodies lack specificity [20,21,25], our experiments used the recently generated and highly specific AT1R antibody, D15136-1-5 (see Supplementary Materials, Figure S2). The specificity of this antibody has been validated by Western blotting, demonstrating the presence of a band at the expected molecular weight of AT1Rs (43 kDa) in skeletal muscles from wild type mice; importantly, this band is absent in muscle fibers from AT1R knock-out animals. Additional support for the specificity of the antibody comes from the observation that the density of the 43 kDa band increases in muscles from transgenic animals that overexpress AT1R in skeletal muscle [25].

To compliment the Western blot assessment of AT1R abundance, our experiments also incorporated a fluorescence-based ligand-binding assay to detect both the location and relative abundance of AT1Rs in muscle fibers. Because commercially available AT1R antibodies lack specificity, AngII binding assays are commonly used as the standard method for measuring AT1R abundance in many tissues including the brain, blood vessels, and kidney. However, to our knowledge, the current study is the first application of an AngII binding assay to determine AT1R abundance in skeletal muscle.

Radioligand-binding assays are considered to be the gold standard for quantifying ligand-receptor interactions [31], and while fluorescent-labeled binding assays are less sensitive than radiolabeled methods, fluorescent assays are reliable, safe, and do not produce radioactive waste [32,33]. Moreover, recent improvements in the fluorescence-based ligand binding assay used in this study has reduced the quenching associated with older methods and improved the accuracy of the assay [27,32,33]. Lastly, while the fluorescent-labeled AngII can also bind to Angiotensin II type 2 receptors (AT2Rs), adult skeletal muscle fibers do not express AT2Rs (reviewed in [34]).

As an additional index of AT1R expression in rat skeletal muscle, we measured AT1R mRNA. Only one housekeeping gene, β-actin, was used for comparisons among tissues. It is possible that relative differences of β-actin exist between diaphragm, soleus, and plantaris which could contribute to our findings. Nonetheless, our results confirm that AT1R mRNA is present in isolated single muscle fibers from the diaphragm, soleus, and plantaris muscles. Note that AT1R mRNA was not measured in the human muscle samples because of limited tissue availability.

Our experiments determined the AT1R abundance in both respiratory and locomotor muscles. The diaphragm is a mixed fiber type muscle that contains all the muscle fiber types found in both rat and human skeletal muscle [35] and has been studied because activation of the RAS promotes diaphragmatic atrophy during prolonged mechanical ventilation [22]. Therefore, determining if diaphragm fibers express AT1R is important in the development of therapies to prevent ventilator-induced diaphragmatic wasting.

To establish if AT1Rs are differentially expressed in locomotor skeletal muscles that differ in muscle fiber type composition, we studied the rat soleus and plantaris muscles. These muscles were selected because the rat soleus muscle is primarily composed of slow (type I) fibers, whereas the plantaris muscle is dominated by fast (type II) fibers [24]. Collectively, the methods employed within our investigation provide a rigorous approach to determining the presence of AT1Rs in skeletal muscle.

### 4.3. Physiological and Clinical Implications of AT1Rs in Skeletal Muscle

Although these experiments are limited by the absence of a direct measure of AT1R function in skeletal muscle, the confirmation that AT1Rs are expressed in skeletal muscle fibers has important implications for future studies seeking to understand the mechanism(s) responsible for AngII-mediated muscle wasting. Explicitly, because of the lack of persuasive evidence that skeletal muscles express AT1R, it has been postulated that AngII-induced muscle atrophy occurs via the indirect effects of AngII acting to increase circulating cortisol or cytokines [10]. However, given the compelling evidence within the current report that AT1Rs are expressed in skeletal muscles, it appears probable that AngII stimulated muscle atrophy can also occur by activation of AT1Rs on the sarcolemma. The link between AT1R activation and muscle atrophy is likely the AT1R-provoked activation of NOX2 and the subsequent production of reactive oxygen species (ROS) [3]. Indeed, it is established that oxidative stress promotes skeletal muscle atrophy by both accelerated proteolysis and depressed protein synthesis [12]. Evidence that AngII-mediated muscle atrophy occurs via NOX2-induced oxidative stress is supported by the observation that knockout mice lacking p47 (phox), a required subunit for NOX2 activation, are protected against AngII-induced muscle wasting [36].

Interestingly, emerging evidence reveals that, independent of elevated circulating AngII, activation of AT1Rs contribute to the diaphragmatic atrophy that occurs during prolonged mechanical ventilation (MV) [22]. Although MV is a life-saving intervention for patients in respiratory failure, an unintended consequence of prolonged MV is the rapid development of diaphragmatic atrophy [37]. Preclinical studies have demonstrated that pharmacological blockade of AT1Rs protects against ventilator-induced diaphragmatic atrophy [22]. In contrast, prevention of the increase in circulating AngII (via pharmacological inhibition of angiotensin converting enzyme) does not avert MV-induced diaphragmatic atrophy [22]. Moreover, plasma levels of cortisol, IL-6, and SAA do not increase during prolonged MV. Therefore, these results confirm that activation of AT1Rs is required for MV-induced diaphragmatic atrophy and that indirect RAS signaling does not contribute to ventilator-induced diaphragmatic atrophy. In this regard, evidence shows that independent of ligand binding, AT1R activation can occur in response to mechanical activation of the receptor [38]. Hence, it is feasible that diaphragmatic AT1Rs are activated during MV by mechanical forces applied to the sarcolemma [38]. Specifically, during MV, diaphragm muscle fibers undergo repetitive, passive length changes (shortening and lengthening cycles); the force of these passive length changes is imposed by transverse forces from the MV-induced (positive pressure) inflation of the lung rather than the axial forces that occur during normal diaphragm contractions [39]. Moreover, for a given change in lung volume, diaphragm fibers shorten more during MV than during spontaneous breathing [3,40]. Hence, it is predicted that the transverse tension on the sarcolemma of diaphragm fibers is greater during MV as compared with spontaneous breathing. If this is the case, this MV-induced increase in membrane tension could be responsible for the activation of AT1Rs in diaphragm fibers [3,41,42].

While preclinical studies provided the first proof that prolonged MV results in rapid diaphragmatic atrophy, it is now established that MV-induced diaphragmatic atrophy also occurs in humans [43,44,45]. MV-induced diaphragmatic atrophy is a serious clinical problem because diaphragm weakness is a major risk factor for problems in weaning patients from the ventilator [46]. Therefore, our discovery that AT1Rs are expressed in human diaphragm fibers is important and provides key insights into a potential biological target for future therapies to prevent MV-induced diaphragmatic atrophy.

In addition to confirming that skeletal muscles express AT1Rs, our results also provide the first evidence that AT1R abundance is higher in skeletal muscles as composed with that of fast-twitch muscle fibers (i.e., plantaris) as compared with muscles comprised of slow fibers (i.e., soleus) and muscles containing a combination of both slow and fast fibers (i.e., diaphragm) [24]. The discovery that AT1Rs are more abundant in fast muscle fibers provides biological insight into a potential mechanism to explain why diseases associated with high circulating levels of AngII (i.e., chronic kidney disease, heart failure, etc.) promote greater atrophy in locomotor muscles comprised of primarily type II fibers [47,48,49]. In a related finding, evidence indicated that NOX2 activity was markedly increased in the plantaris muscle of rodents in heart failure [50]. This observation raises the intriguing question of whether this heart failure-induced activation of NOX2 was mechanistically linked to the high expression of AT1Rs in the plantaris muscle. Notably, further experiments are required in order to determine if higher AT1R abundance in plantaris translates to increased NOX2 signaling. Indeed, the discovery that AT1R expression is greater in fast muscle fibers should stimulate future research into the mechanisms responsible for the differential rate of skeletal muscle atrophy in diseases associated with high plasma levels of AngII.

## 5. Conclusions

Our results provide the first robust proof that both human and rodent skeletal muscles express AT1Rs. Importantly, our data also deliver the first evidence that AT1R abundance is greater in muscles with a high percentage of type II (fast) fibers as compared with muscles with a high composition of type I (slow) fibers. Historically, physiologists have believed that the primary role of the RAS is to regulate fluid, electrolyte, and blood pressure homeostasis. However, our findings that AT1Rs exist in skeletal muscle raise important questions that should stimulate future research on the physiological function of AT1Rs in both healthy skeletal muscle and in muscles undergoing atrophy in response to both disease and muscle inactivity (e.g., heart failure, kidney disease, or prolonged mechanical ventilation). Lastly, the discovery that AT1Rs exist in the human diaphragm is an important and novel finding that has significant potential to contribute to future research into the involvement of AT1Rs in ventilator-induced diaphragmatic atrophy and the development of protective therapies.

## Supplementary Materials

The following are available online at https://www.mdpi.com/2073-4409/9/7/1688/s1, Figure S1: In vitro localization of AT1R in smooth muscle of rat aorta (vasculature), renal cortex and plantaris muscle, Figure S2: Full length images of western blots for AT1R and total protein in rodent and human muscle.
