# Supplementary material for: Human and Rodent Skeletal Muscles Express Angiotensin II Type 1 Receptors
Source: Cells. 2020 Jul 14;9(7):1688. doi: 10.3390/cells9071688 (PMC7407103; doi:10.3390/cells9071688)
Supplement: Supplementary file 1 [file cells-09-01688-s001.pdf]

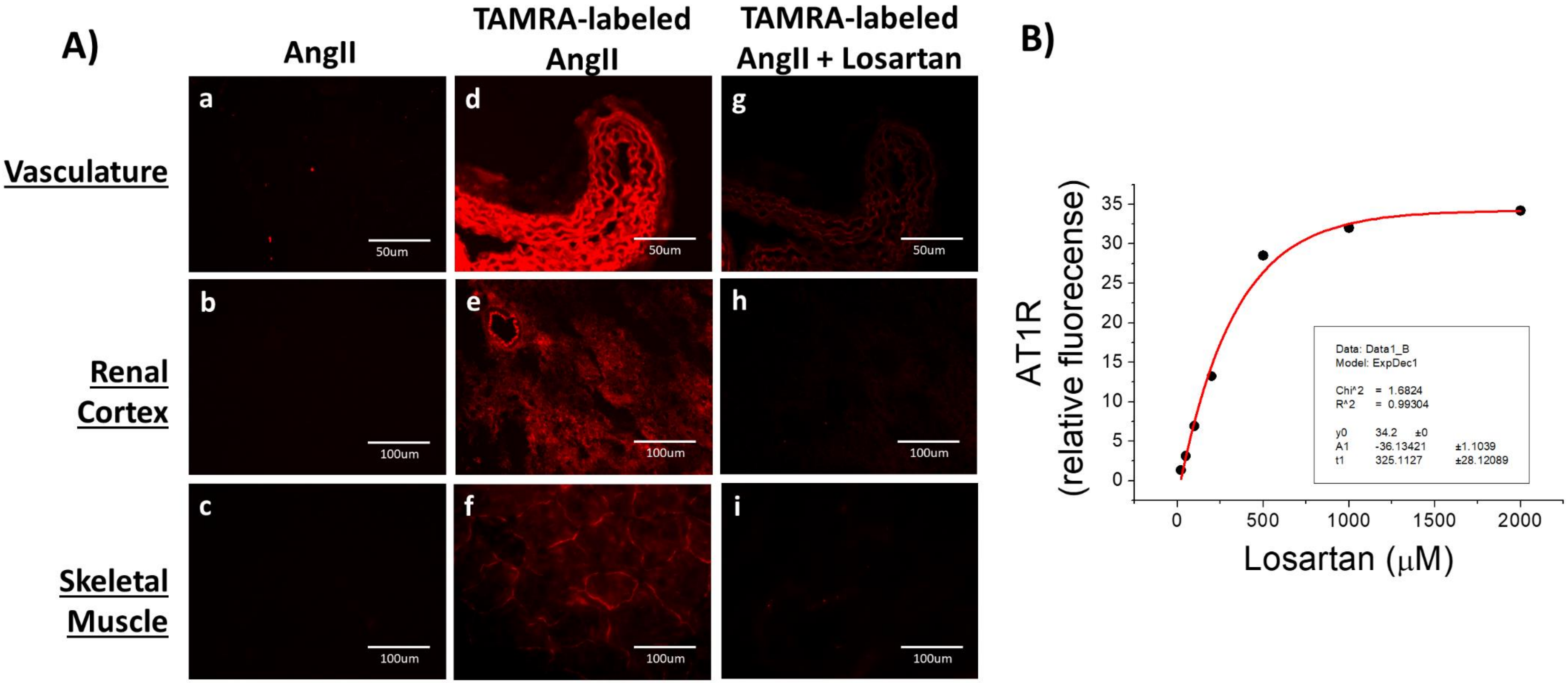

Supplementary Figure 1. In vitro localization of AT1R in smooth muscle of rat aorta (vasculature), renal cortex and plantaris muscle (**A**). Figure demonstrates absence of fluorescence signal after incubation with non-labeled Ang II (a, b and c), total binding after incubation with TAMRA-labeled Ang II (d, e and f) and nonspecific binding after incubation with TAMRA-labeled Angiotensin II + 1 mM losartan (g, h and i). Dose-response curve demonstrated 1mM losartan promoted maximal AT1R inhibitory effect on plantaris skeletal muscle (**B**).

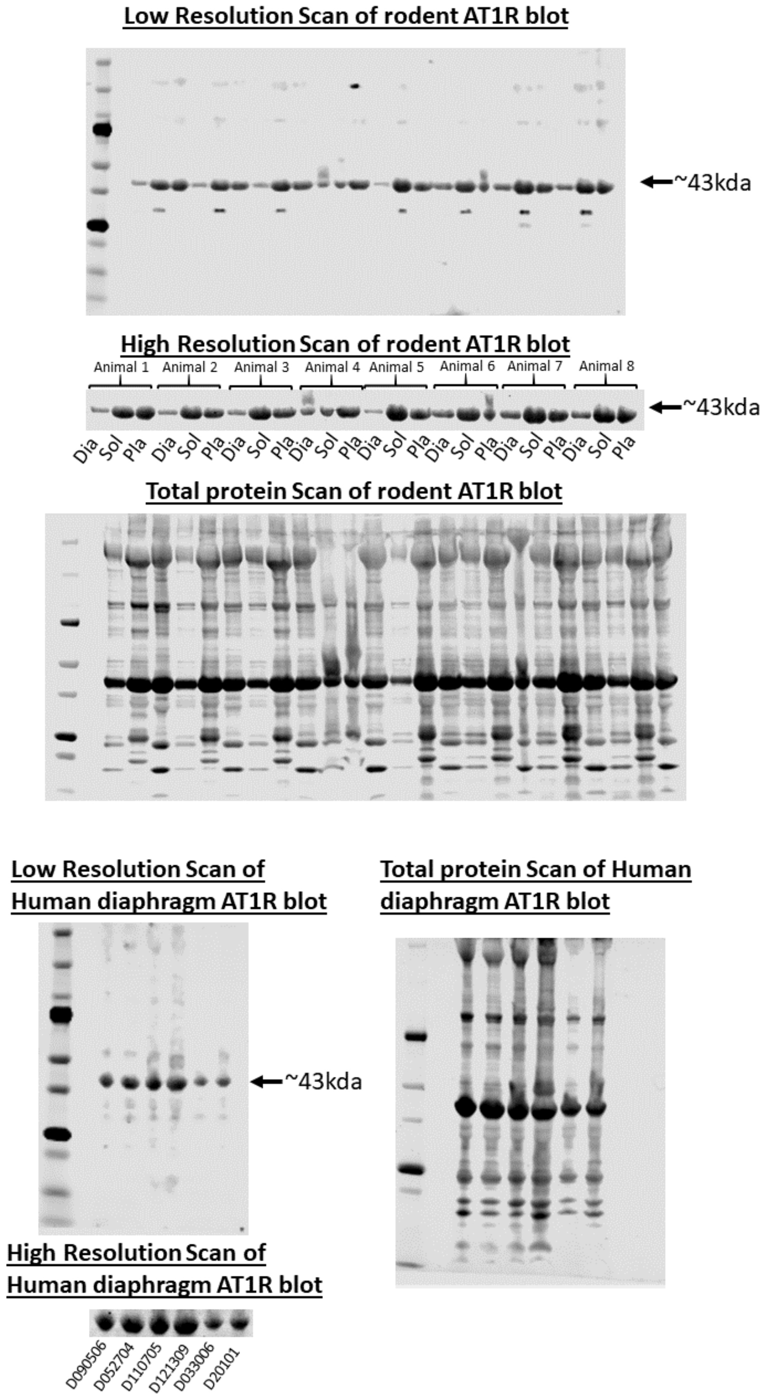

Supplementary Figure 2. Full length images of western blots for AT1R and total protein in rodent and human muscle.
